# Skeletal Muscle Loss during Multikinase Inhibitors Therapy: Molecular Pathways, Clinical Implications, and Nutritional Challenges

**DOI:** 10.3390/nu12103101

**Published:** 2020-10-12

**Authors:** Emanuele Rinninella, Marco Cintoni, Pauline Raoul, Carmelo Pozzo, Antonia Strippoli, Francesca Romana Ponziani, Maurizio Pompili, Emilio Bria, Giampaolo Tortora, Antonio Gasbarrini, Maria Cristina Mele

**Affiliations:** 1 UOC di Nutrizione Clinica, Dipartimento di Scienze Mediche e Chirurgiche, Fondazione Policlinico Universitario A. Gemelli IRCCS, Largo A. Gemelli 8, 00168 Rome, Italy; 2 Scuola di Specializzazione in Scienza dell’Alimentazione, Università di Roma Tor Vergata, Via Montpellier 1, 00133 Rome, Italy; marco.cintoni@gmail.com; 3 UOSD di Nutrizione Avanzata in Oncologia, Dipartimento di Scienze Mediche e Chirurgiche, Fondazione Policlinico Universitario A. Gemelli IRCCS, Largo A. Gemelli 8, 00168 Rome, Italy; pauline.raoul1@gmail.com (P.R.); mariacristina.mele@unicatt.it (M.C.M.); 4 Comprehensive Cancer Center, Fondazione Policlinico Universitario A. Gemelli IRCCS, Largo A. Gemelli 8, 00168 Rome, Italy; carmelo.pozzo@policlinicogemelli.it (C.P.); antonia.strippoli@policlinicogemelli.it (A.S.); emilio.bria@unicatt.it (E.B.); giampaolo.tortora@policlinicogemelli.it (G.T.); 5 UOC di Medicina Interna e Gastroenterologia, Dipartimento di Scienze Mediche e Chirurgiche, Fondazione Policlinico Universitario A. Gemelli IRCCS, Largo A. Gemelli 8, 00168 Rome, Italy; francesca.ponziani@yahoo.it (F.R.P.); maurizio.pompili@unicatt.it (M.P.); antonio.gasbarrini@unicatt.it (A.G.); 6 Dipartimento di Medicina e Chirurgia traslazionale, Università Cattolica Del Sacro Cuore, Largo F. Vito 1, 00168 Rome, Italy

**Keywords:** muscle mass, sarcopenia, sorafenib, regorafenib, lenvatinib, sunitinib, toxicity, survival, nutrition, personalized medicine

## Abstract

In cancer patients, loss of muscle mass is significantly associated with low tolerability of chemotherapy and poor survival. Despite the great strides in the treatment of cancer, targeted therapies such as tyrosine kinase inhibitors (TKIs) could exacerbate muscle wasting. Over recent years, the impact of skeletal muscle loss during TKI therapy on clinical outcomes has been in the spotlight. In this review, we focus on the different molecular pathways of TKIs potentially involved in muscle wasting. Then, we report the results of the studies assessing the effects of different TKI therapies—such as sorafenib, regorafenib, sunitinib, and lenvatinib—on muscle mass, and highlight their potential clinical implications. Finally, we discuss an integrative nutritional approach to be adopted during TKI treatment. The assessment of muscle mass from computerized tomography imaging could be helpful in predicting toxicity and prognosis in patients treated with TKI such as sorafenib. Early recognition of low muscle mass and effective personalized nutritional support could prevent or attenuate muscle mass wasting. However, the role of nutrition is still overlooked, and future clinical trials are needed to find the optimal nutritional support to countermeasure muscle mass depletion during TKI therapy.

## 1. Introduction

The discovery of the overexpression of kinases in various cancers has led to the development of tyrosine kinase inhibitors (TKIs). TKIs demonstrated to produce a significant improvement in survival rates in several cancers. Just to name a few examples, imatinib has revolutionized the treatment of chronic myelogenous leukemia as well as gastrointestinal stromal tumors (GISTs) [1]; sorafenib was the first therapy proven to prolong survival in patients with metastatic hepatocellular carcinoma (HCC) [2]; sunitinib provided for the first time a survival advantage over interferon to treat metastatic renal cell cancer (RCC) [3]. However, despite the great strides in the treatment of cancer, these agents can also induce frustrating dose-limiting toxicities (DLTs) partially due to the loss of body weight and muscle mass [4]. Indeed, the reduction in physical activity, the nutritional deficiencies resulting from cancer itself, and the side effects of oncologic treatment—such as nausea, vomiting, diarrhea, taste alteration, and early satiety—often lead to a loss of weight and lean tissue mass. Patients with poor nutritional status are often not able to tolerate chemotherapy and discontinue treatment. Low skeletal muscle mass was already shown to be a significant predictor of chemotherapy toxicity and survival in cancer patients [5,6,7]. Thus, research increasingly focuses on the importance of preserving skeletal muscle mass of patients receiving chemotherapy. In recent decades, a growing number of studies attempted to understand whether muscle wasting was exacerbated by TKI treatment and if such muscle loss was associated with toxicity and survival outcomes. This review aims to understand how TKI therapy could impact muscle mass in cancer patients and highlights potential clinical implications and nutritional opportunities. After focusing on the specific molecular pathways of TKI involved in muscle wasting, we review the impact of TKI therapy on muscle mass for several types of cancer and its implications in terms of clinical outcomes. Finally, we discuss the state of the art about nutritional strategies to counteract muscle wasting during these treatments.

## 2. Molecular Pathways of TKI Involved in Muscle Wasting

The maintenance of skeletal muscle mass is determined by a close balance between protein synthesis and protein degradation. Intracellular signaling cascades, regulating the mechanisms for muscle growth or muscle loss, initiate with a variety of chemical signals depending on nutritional and hormonal status—such as insulin or insulin-like growth factor-1 (IGF-1)—or energy state (AMP kinase) and physical activity, or other mediators of environmental stress (glucocorticoids, cytokines). A key point of integration in muscle protein synthesis is the phosphoinositide 3-kinase (PI3K)/thymoma viral proto-oncogene (AKT) kinase [8]. The insulin/IGF-1- Akt pathway increases skeletal muscle protein synthesis via inhibiting glycogen synthase kinase 3β and activating the mechanistic target of rapamycin complex 1 (mTORC1) signaling [9]. In turn, mTORC1 activates translation initiation and elongation, and ribosome biogenesis of proteins, and consequently muscle cell growth [10].

Tyrosine kinases are enzymes that target proteins involved in diverse normal cellular regulatory processes [11]. The receptors of tyrosine kinases are membrane-spanning cell surface proteins linking extracellular signals to the cytoplasm [12]. Ligand binding induces dimerization of these receptors, resulting in autophosphorylation of their cytoplasmic domains and activation of tyrosine kinase activity [11]. TKIs target different receptor tyrosine kinases such as vascular endothelial growth factor receptors (VEGFRs). VEGFRs 1/2/3 are located on vascular endothelial cells and play a key role in angiogenesis [13]. In healthy adults, angiogenesis is a complex multistep process that is tightly controlled by a balance between endogenous pro-angiogenic and anti-angiogenic factors. However, angiogenesis is a crucial prognostic factor in cancer and frequently correlates with tumor progression, disease severity, and metastatic potential [14]. TKIs such as sorafenib and sunitinib partly exert their anti-tumor activity by inhibiting the tyrosine kinase activity of VEGFR-2 [15]. Platelet-derived growth factor receptors (PDGFRs) are also TKIs’ targets involved in angiogenesis: mutational activation or upregulation of PDGFRs lead to uncontrolled blood vessel formation and cancer. Moreover, PDGFR-β emerged as a key regulator of cell growth and division and mediates a significant impact on malignant cells and tumor microenvironment [16]. Epidermal growth factor receptors (EGFRs) are transmembrane protein receptors for extracellular protein ligands belonging to the group of epidermal growth factor (EGF) [17]. The overexpression of the EGFR is associated with the development of a wide variety of tumors such as non-small cell lung cancer and breast cancer [18]. Finally, KIT, RET, and B-RAF are other receptor tyrosine kinases encoded by proto-oncogenes: the overexpression or mutations of these proteins can promote carcinogenesis in several tissues [19]. 

**Figure 1:**
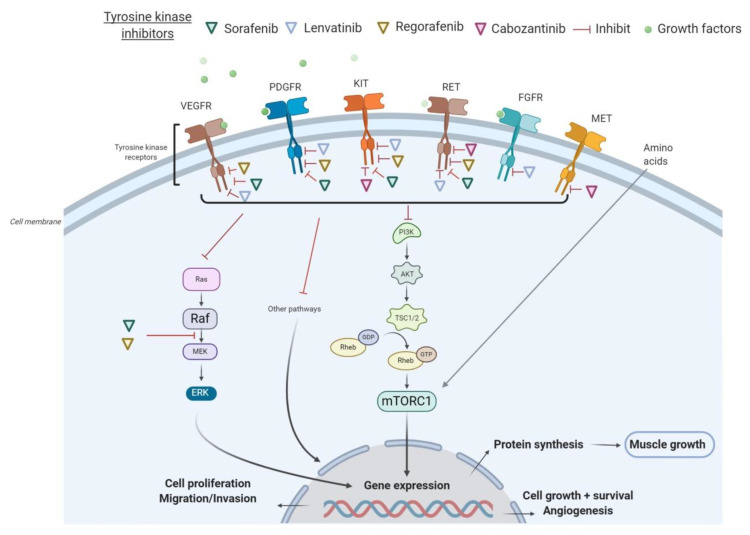
Mechanisms of action of tyrosine kinase inhibitors (TKIs) in hepatocellular carcinoma (HCC). Abbreviations: FGFR, fibroblast growth factor receptors; GDP, guanosine diphosphate; GTP, guanosine triphosphate; mTORC1, mammalian target of rapamycin complex 1; PDGFR, platelet-derived growth factor receptor; PI3K, phosphoinositide 3-kinase; TSC, tuberous sclerosis complex; VEGFR, vascular endothelial growth factor receptor.

All these tyrosine kinase receptors have been associated with the signaling pathway of activation of the PI3K/AKT/mTOR, which promotes cell growth, survival, and proliferation. In many cancers, the PI3K/AKT/mTOR pathway is overactive, thus reducing apoptosis and allowing cell proliferation [20]. On the other hand, the activation of the AKT/mTOR pathway and its downstream targets is essential for regulating skeletal muscle fiber size [21]. In vivo, genetic activation of the AKT/mTOR pathway caused muscle hypertrophy and prevented atrophy, whereas blocking of this pathway blocked muscle hypertrophy [22]. By inhibiting receptor tyrosine kinase signaling, TKIs have shown to indirectly suppress AKT and mTOR. In particular, sorafenib blocks VEGFR, PDGFR, c-KIT, and RET and inhibits downstream Raf serine/threonine kinase activity to prevent tumor growth, as demonstrated in advanced HCC, RCC, and unresectable thyroid cancer [23]. Additionally, regorafenib is an oral multikinase inhibitor of VEGFR1-3, KIT, RET, B-RAF, and PDGFR [24]. As illustrated in HCC in Figure 1, several TKIs indirectly inhibit mTOR and consequently impair cell proliferation, protein synthesis, and muscle growth.

Although the effects of mTOR inhibitors on muscle mass have still to be fully elucidated, a recent human study [25] demonstrated that the long-term use of mTOR inhibitors induces a marked loss of muscle mass. This confirms that TKIs may alter muscle mass, probably due to interferences with pathways of AKT/mTOR. As reported in the next paragraph, several studies assessed the impact of TKI treatment on muscle mass in cancer patients.

## 3. Impact of TKI Treatment on Muscle Wasting

An electronic search was conducted in PubMed and Web of Science databases, using different combinations of the search terms “(Afatinib OR Alectinib OR Axitinib OR Bosutinib OR Brigatinib OR Cabozantinib OR Ceritinib OR Crizotinib OR Dasatinib OR Erlotinib OR Gefitinib OR Ibrutinib OR Imatinib OR Lapatinib OR Lenvatinib OR Nilotinib OR Osimertinib OR Pazopanib OR Ponatinib OR Regorafenib OR Ruxolitinib OR Sorafenib OR Sunitinib OR Vandetanib) AND (sarcopenia OR (muscle OR muscular *) OR (body AND composition))”. The screening process was conducted independently by two reviewers. Any disagreements were solved through discussion until consensus. Our search was conducted until July 2020. Table 1 detailed the results of the eleven studies [26,27,28,29,30,31,32,33,34,35,36] assessing the effect of TKI treatment on skeletal muscle mass from computerized tomography (CT) scan at the third lumbar (L3) vertebra. 

### 3.1. Sorafenib

Sorafenib is approved for the treatment of advanced HCC, metastatic RCC, and unresectable thyroid cancer [37]. Sorafenib blocks VEGFR, PDGFR, c-KIT, and RET, and inhibits downstream Raf serine/threonine kinase activity to prevent tumor growth [38].

Antoun et al. [31] studied muscle mass changes in 48 patients with metastatic RCC treated with sorafenib. They noted that 52% of patients had low skeletal muscle index (SMI) before initiation of treatment with sorafenib (for men < 52.4 cm^2^/m^2^ and for women < 38.5 cm^2^/m^2^), whereas after one year of treatment, 71% of patients had low SMI, with an average weight loss of 4.2 kg. Another study [34] evaluated 101 metastatic RCC patients including 45 patients treated with sorafenib and 30 with sunitinib. The mean SMI was reduced from 41.6 to 39.9 cm^2^/m^2^ after 3–4 months of targeted therapy. Furthermore, Uchikawa et al. evaluated 23 HCC patients treated with sorafenib and found a significant decrease in SMI between baseline and 1–3 months after starting treatment (*p* < 0.01) [33]. To better investigate the role of the treatment itself in muscle wasting, a post hoc analysis of the Phase III DECISION trial study [32] compared SMI changes among 365 patients with advanced thyroid cancer in sorafenib vs. placebo groups between baseline and 6 months after starting sorafenib therapy. At 6 months, the mean muscle mass of patients receiving sorafenib was lower than at baseline and significantly lower than for patients receiving placebo (*p*  <  0.0001). 

Although the mechanisms of sorafenib remain poorly understood, these findings could be partially explained by the inhibition of the Ras/Raf/MEK/extracellular signal-regulated kinase (ERK) signaling pathway that, in turn, inhibits muscle anabolism [39]. Moreover, the inhibition of VEGF expression by sorafenib may occur also via the ERK/nuclear factor (NF)-κB pathway to reduce metastasis and invasiveness of human HCC [40]. The NF-κB signaling pathway interestingly impacts both the muscle fibers and muscle stem cells [41]. These mechanisms of sorafenib—like other TKIs sharing the same molecular targets—may have a possible relationship with muscle wasting. However, these conjectures need to be confirmed with further experimental studies.

### 3.2. Lenvatinib

Lenvatinib is an oral multikinase inhibitor targeting VEGF1–3, fibroblast growth factor receptors 1–4 (FGFR1–4), RET, c-KIT, stem cell growth factor receptor (SCGFR), and PDGFRα [42]. It is approved as first-line treatment for advanced and metastatic differentiated thyroid carcinoma after radioactive iodine failure and for the treatment of advanced HCC in first-line therapy [43]. Recently, a brief report of two case reports of patients with advanced unresectable HCC treated with lenvatinib found a minimal impact of lenvatinib on muscle mass—detected with CT scan—with stable disease for over 24 months [27]. On the other hand, Hiraoka et al. reported a post hoc analysis of a multicentre study quantifying psoas muscle loss by CT scan in HCC patients treated with lenvatinib and demonstrated a decline in PI after 4 weeks of treatment (−0.210 ± 0.315 cm^2^/m^2^) in 41 patients and after 12 weeks of treatment (−0.275 ± 0.372 cm^2^/m^2^) in 25 patients [28]. However, Hiraoka et al. used psoas muscle assessment, which is less precise and suitable to assess total muscle mass than skeletal muscle indexes such as SMI and skeletal muscle area (SMA) (see above). Recently, a subgroup analysis of the study of Uchikawa et al. found in 8 patients (with advanced HCC and Child-Pugh grade A) treated with lenvatinib a significant decrease in SMI between baseline and 1–3 months after starting treatment (*p* = 0.025) [33], independently with progression disease. Further studies with a larger sample size are needed to clarify the impact of lenvatinib on muscle mass. 

### 3.3. Sunitinib

Sunitinib is a multitargeted TKI, inhibiting PDGFRs, KIT, and VEGFR-1-2-3 [44]. Sunitinib is the reference standard of care for the first-line treatment of metastatic RCC or pancreatic neuroendocrine tumors (PNETs), and for the second-line treatment for patients affected by unresectable and/or metastatic GISTs who had a failure to imatinib. One study found a significant decrease in lean body mass (LBM) (*p* = 0.02) [35] and skeletal muscle area SMA (*p* = 0.02) in 18 metastatic RCC patients after starting sunitinib treatment (3–4 months) compared with baseline. Gu et al. [34] also found in 30 (out of 101) patients treated with sunitinib a reduction in the mean SMI from 41.6 to 39.9 cm^2^/m^2^ after 3–4 months of therapy. 

### 3.4. Regorafenib

Regorafenib is an oral multikinase inhibitor, pharmacologically similar to sorafenib, that blocks kinases involved in angiogenesis (VEGFR-1-2-3), oncogenesis (c-KIT, Ret and wild-type, and V600-mutated BRAF), and the tumor microenvironment (PDGFR and FGFR) [38]. Regorafenib is, to date, approved for the second-line treatment of metastatic colorectal cancer (CRC), after the failure of fluoropyrimidine-, oxaliplatin-, and irinotecan-based chemotherapy; moreover, it is effective in second-line therapy for the treatment of advanced HCC after sorafenib failure [45], and in patients affected by GISTs who had progression or intolerance to imatinib or sunitinib. 

In an animal study, regorafenib has been shown to cause skeletal muscle loss through a possible mechanism including increasing levels of autophagy-dependent protein markers and abnormal mitochondrial homeostasis via ERK1/2 and glycogen synthase kinase 3 beta (GSK3β) pathways [46]. In humans, one study [29], evaluating 22 CRC patients treated with regorafenib, found a statistically significant skeletal muscle loss during treatment (median SMI change: −2.75 cm^2^/m^2^; *p* < 0.0001). Furthermore, a recent study assessed skeletal muscle mass (SMM) changes in 36 metastatic CRC patients, who received regorafenib or TAS-102 (a novel oral fluoropyrimidine) in third-line treatment [30]. The SMM change after regorafenib therapy was significantly worse compared with TAS-102 therapy (*p* = 0.001) [30].

### 3.5. Pazopanib

Pazopanib is a multikinase inhibitor approved for the treatment of patients with advanced RCC or advanced soft tissue sarcoma, in the second-line [47]. Pazopanib is a VEGFR inhibitor with activity against vascular EGFR-1, -2, and -3, and PDGFRs [48]. Kostek et al. [35] found, in 18 metastatic RCC patients, a non-significant decrease in LBM and SMA after starting pazopanib. Conversely, they noted that SMA and LBM were significantly reduced with sunitinib therapy compared with pazopanib therapy. The reason for these discrepancies is unclear, but it might be related to their different kinase selectivities [35]. Indeed, sunitinib was shown to inhibit more kinases than pazopanib [49]. However, further studies are warranted to analyze the effects of pazopanib on the muscle mass and the implications for clinical outcomes. 

### 3.6. Axitinib

Axitinib mostly targets VEGFR-1-2-3 [50]. It is indicated for the treatment of advanced RCC after failure of one prior systemic therapy (sunitinib or cytokines) [51]. One study [26] evaluated the effect of axitinib on muscle mass in 24 patients with advanced non-metastatic RCC (*n* = 23), showing a significant decrease in SMI at 12 weeks after starting treatment compared to baseline (−2.9 cm^2^/m^2^; IQR 1.7–6.2; *p* < 0.001).

### 3.7. Vandetanib

Vandetanib selectively blocks EGFR and VEGFR-2. It is approved for the treatment of symptomatic or progressive medullary thyroid cancer in patients with unresectable or metastatic disease [52]. A randomized controlled trial of 33 patients with advanced medullary thyroid carcinoma compared SMI changes from baseline to month 3 after starting treatment in patients treated with vandetanib vs. patients treated with placebo [36]. Surprisingly, vandetanib was found to be significantly associated with a gain in muscle mass over time (*p* = 0.009). The authors hypothesized that vandetanib, by inhibiting the MAPK pathway, could reduce the burst of inflammatory cytokines—especially interleukin (IL) 6—involved in the stimulation of muscle growth and myogenesis through regulation of the proliferative capacity of muscle stem cells [53]. Another protective mechanism could be the reduction in diarrhea during vandetanib therapy. In any case, further studies are required to confirm these results.

Considering these preliminary findings and the negative prognostic role of low muscle mass in cancer, muscle wasting during multikinase inhibitor therapy may impact clinical outcomes. Several studies further assessed the associations between muscle mass changes during TKI treatment and toxicity and/or survival.

## 4. Implications of Muscle Wasting in Clinical Outcomes in Patients Treated with TKI 

In this part, we highlight the impact of low muscle mass/muscle wasting on clinical outcomes during TKI treatment for several types of cancer.

### 4.1. RCC 

A recent study found that RCC patients treated with axitinib with baseline low SMI tended to have a lower response rate to treatment [26]. Moreover, low SMI is predictive of treatment-related toxicity in patients with metastatic RCC receiving sunitinib [54] or sorafenib [31,55]. Particularly, Kostek et al. noted that loss of SMA and LBM with sunitinib was more substantial than with pazopanib [35]. Although treatment efficacies of both drugs were similar, DLTs were more frequent in RCC patients treated with sunitinib compared with pazopanib. Similarly, muscle wasting during TKI treatment could have a potential impact on survival outcomes. Patients with metastatic RCC and low SMI have been shown to have a worse overall survival (OS) after cytoreductive nephrectomy compared with patients with high SMI [56]. Fukushima et al. also showed that low SMI was significantly associated with worse OS in 92 patients with metastatic RCC [57]. 

### 4.2. HCC 

In HCC patients receiving sorafenib, Mir et al. showed that the amount of DLTs was significantly correlated with low SMI [4]. Furthermore, a cohort study of European Caucasian cirrhotic patients with advanced HCC treated with sorafenib showed that low SMI was a predictor of reduced survival and cancer treatment toxicity [58]. Recently, Uojima et al. showed the number of severe adverse advents during the first two months of lenvatinib treatment was significantly associated with low SMI in 100 patients with HCC [59]. Low muscle mass has been already demonstrated to be a strong and independent risk factor for mortality in advanced HCC patients [60]. Consequently, we can infer that muscle wasting during treatment induced by sorafenib or lenvatinib therapy could negatively impact toxicity and survival in HCC patients. Moreover, the alterations of the nutritional status by TKI treatment and HCC itself could be also incremented by the underlying disease, such as liver cirrhosis [61]. Indeed, in cirrhosis, malnutrition may be due to several conditions: pancreatic insufficiency, cholestasis, portosystemic shunt, bile deficiency through inadequate absorption of long-chain fatty acids, metabolic alterations such as high protein catabolism, reduced glucose homeostasis due to alterations of gluconeogenesis, low glycogen stores, pro-inflammatory cytokines such as TNF alpha, interleukins [62]. Therefore, considering the high prevalence of sarcopenia in HCC patients and the effects of TKI shown on muscle mass, more attention on muscle mass changes during therapy is needed when TKIs are prescribed. 

### 4.3. CRC 

Low muscle mass is present in up to 40% of patients at the initial metastatic CRC diagnosis [63]. Moreover, several studies [64,65] found a loss of skeletal muscle during chemotherapy in patients with metastatic CRC and its association with treatment modifications, toxicity, and survival. Recently, Gokyer et al. assessed a significant association between low SMI and DLT in 36 patients with metastatic CRC who received regorafenib [66]. In this study, they determined more DLTs (*p* = 0.005) and grade 2 or 3 toxicities in low SMI patients compared with the high SMI group. However, there were no significant differences in terms of progression-free survival (PFS) and OS between sarcopenic and non-sarcopenic patients. More recently, Bekir et al. showed in a Cox regression analysis that loss of skeletal muscle mass was an independent prognostic indicator for OS (HR, 2.87; 95%CI: 1.07–7.42; *p* = 0.03) in 36 metastatic CRC patients [30]. These results confirmed that in clinical practice, CRC patients with low muscle mass should be detected and followed at the beginning of the treatment. 

### 4.4. Thyroid Cancer

Despite a significant effect of sorafenib on muscle mass, the risk of early toxicity leading to dose modification was not significantly higher in thyroid cancer patients with low SMI compared with those with high SMI in a study of Huillard et al. [32]. These results contrast with those in HCC and RCC patients. Discrepancies between cancer types could be explained by the differences in underlying disease and the effects of prior treatments. Indeed, patients with RCC may have been treated also with interferon, a pro-inflammatory cytokine that has complex physiologic effects, including effects on skeletal muscle homeostasis and repair. In HCC, muscle mass depletion is a common feature of cirrhosis, commonly found in patients with HCC, representing an independent prognostic factor. 

Although these findings need to be confirmed, given that muscle wasting could be exacerbated by TKI treatment and associated with worse clinical outcomes, monitoring muscle mass changes during TKI treatment and proposing adequate nutritional support could represent an opportunity to prevent or treat low muscle mass during TKI therapy, leading to better treatment tolerability and clinical outcomes.

## 5. Nutritional Challenges 

### 5.1. Early Assessment and Monitoring of Nutritional Status During Treatment

As detailed in Table 1, many cancer patients undergoing TKI therapy are already sarcopenic before the initiation of treatment, with a percentage ranging from 30.4% to 54%. Sarcopenia is a condition characterized by a loss of skeletal muscle mass quantity, quality, and function. The European Working Group on Sarcopenia in Older People 2 (EWGSOP2) defined sarcopenia as primary when no specific cause is evident except for age. Indeed, aging is associated with hormone and cytokine imbalance, impaired muscle protein synthesis and regeneration, and increased splanchnic extraction (the retention of dietary amino acids by the gut and liver for their own needs) which lower the anabolic response to ingested proteins [67]. Sarcopenia is considered secondary when other causes are evident such as illness, physical inactivity, or inadequate intake of energy due to anorexia or malabsorption [68]. Cancer-related sarcopenia is recognized by international consensus as a negative prognostic factor for OS, complications after surgery, and toxicities in cancer patients. Cancer itself activates a systemic inflammation syndrome, with impaired protein turnover, anabolic resistance, loss of muscle mass, and acute-phase protein synthesis. Moreover, cancer patients often reduce food intake due to primary anorexia (sustained by the effect of cancer cytokines at the central nervous system level) and physical impairment of the digestive system (xerostomia, nausea, vomiting, diarrhea, intestinal obstruction, or malabsorption) [69]. Furthermore, hospitalization and bed-rest are associated with a significant decline in muscle mass and nutritional status [70,71]. 

As regards the studies reported in this review, the median age varied from 51 from 72 years: thus, muscle wasting could be also related to age-related anabolic resistance. However, studies assessed the effect on muscle changes from baseline to approximately 3–12 months of TKI treatment. Considering the short duration of treatment, the effect of age-related muscle loss could be reasonably overlooked as a confounding factor.

In this context, international guidelines [72] recommend the screening and assessment of the nutritional status of cancer patients at diagnosis with the evaluation of nutritional intake, weight changes, and body mass index (BMI). The most commonly used screening tools are Nutrition Risk Screening 2002 (NRS-2002), and Malnutrition Universal Screening Tool (MUST). Although such methods are standardized and easy to perform by healthcare staff—without specific nutritional skills—the assessment of nutritional status is still underused in oncology. In patients identified at high risk of malnutrition, objective and quantitative assessment of muscle mass, physical performance, and degree of systemic inflammation are strongly recommended by the European Society for Clinical Nutrition and Metabolism (ESPEN) guidelines [72]. Such evaluations are performed by clinical nutritionists and trained dietitians. Regarding muscle mass, a severe depletion is assessed by validated methods, such as

− Appendicular skeletal muscle index (ASMI), determined by dual-energy x-ray absorptiometry (men < 7.26 kg/m^2^; women < 5.45 kg/m^2^);
− SMI, determined from oncological CT imaging (men < 55 cm^2^/m^2^; women < 39 cm^2^/m^2^);
− Whole body fat-free mass index without bone determined by bioelectrical impedance (men < 14.6 kg/m^2^; women < 11.4 kg/m^2^).

As we previously described, we systematically searched studies assessing muscle mass during TKI therapy. All studies assessing the effects of TKI treatment on skeletal muscle mass used computerized tomography (CT) image analysis at the third lumbar level (L3). CT scan is, to date, considered the gold standard non-invasive tool to assess muscle mass quantity and quality [73]. CT-scan images are always available for cancer patients since CT is routinely used at diagnosis for tumor staging and at follow-up visits to monitor response to treatments. Some studies [27,30,31] assessed SMA, evaluating all the cross-sectional skeletal muscle area at the third lumbar vertebra (bilateral rectus abdominis, external oblique, internal oblique, transverse abdominal, psoas, quadratus lumborum, and paraspinal muscle). Other studies calculated SMI by dividing SMA by the square of the height [26,29,33,34,36] or skeletal muscle density (SMD; in Hounsfield) [34] or LBM estimated from SMA as described by Mourtzakis et al. [73]. One study [28] used the bilateral psoas index (PI; in cm^2^/m^2^) by dividing psoas muscle area by the square of the height. However, the psoas is a minor muscle, and experts argue that it is not representative of overall sarcopenia [74,75].

Since the majority of patients experienced a loss of muscle mass during their disease course before initiation of TKI therapy, early assessment and reassessment of skeletal muscle mass during treatment should be performed to screen patients and to propose adequate nutritional support.

### 5.2. Personalized Nutritional Support

To date, as far as we are aware, no data are available on nutritional interventions and muscle mass in cancer patients treated with TKIs. Hence, we need to refer to standard guidelines for nutrition in cancer patients. The ESPEN guidelines strongly recommended a personalized nutritional support with specific energy (between 25 and 30 kcal/kg/day) and protein (above 1 g/kg/day and, if possible, up to 1.5 g/kg/day) requirements [72]. Accordingly, oral nutritional supplements (ONS) and artificial nutrition (enteral or parenteral) may be used if oral dietary intake is not sufficient. A recent large, multicenter randomized controlled clinical trial (EFFORT trial) [76] of 2088 medical inpatients at risk of malnutrition evaluated the effects of a protocol-guided individualized nutritional support—including ONS and artificial nutrition—to reach protein and caloric goals on the risk of adverse clinical outcomes. During the hospital stay, caloric and protein goals were, respectively, reached by 79%, and 76% of patients receiving nutritional support. After 30 days, these patients significantly experienced lower rates of adverse events and mortality compared with the control group. Even if cancer patients were only 20% of the entire study population, these findings are the testament of the effectiveness of individualized nutritional support on short-term outcomes in patients at risk of malnutrition [76]. A secondary analysis of the EFFORT trial further showed that there was no legacy effect on long-term outcomes such as mortality at 6 months [77]. This could be explained by the short duration of the length of hospital stay (only 10 days) and consequently too short a time for nutritional support. Thus, there could be a strong rationale to offer continued nutritional support after discharge to reduce high malnutrition-associated mortality. Other works conducted in cancer patients confirm the crucial role of personalized nutritional support. Particularly, in colon and liver cancer patients, the NutriCatt protocol—a nutritional prehabilitation to cancer surgery based on the ESPEN guidelines—ameliorated postoperative complications, length of stay, and hospital costs [78,79]. 

In cancer patients treated with TKIs, a recent observational study [80] showed that energy intake was lower than recommended by the ESPEN guidelines, and none of the patients covered the protein requirements during follow-up. These results highlighted the lack of nutritional assessment and support in multidisciplinary protocols, especially in cancer patients undergoing TKIs treatments.

Among other possible nutritional strategies, branched-chain amino acids (BCAAs) may reduce muscle mass loss in cancer patients. BCAAs are three amino acids with branched side chains (i.e., valine, leucine, and isoleucine). The ability of amino acids to stimulate protein synthesis is reduced in cancer patients. This anabolic resistance could be in a part counteracted by giving specific amino acids [81]. BCAAs have been shown to promote protein synthesis in muscle tissue in rats [82,83]. However, regulation of muscle protein synthesis in rats may differ in many respects compared with humans. Skeletal muscle comprises a much smaller percentage of the total body mass in rats as compared to humans [84]. Nevertheless, in humans, Nair et al. demonstrated that an intravenous infusion of leucine may decrease protein degradation, contributing to the decrease in plasma essential amino acids [85]. In patients with advanced abdominal metastatic adenocarcinoma [86,87], BCAA-enriched formulas may improve whole-body leucine kinetics and leucine balance, thereby favorably influencing protein metabolism in cancer cachexia. A study of Takeda et al. [88] enrolled 78 HCC patients treated with sorafenib into two groups: BCAA granules (Livact; Ajinomoto, Tokyo, Japan) containing 952 mg of L-isoleucine, 1904 mg of L-leucine, and 1144 mg of L-valine per sachet (intervention group) or only a regular diet (control group). In a multivariate analysis, the administration period of sorafenib, as well as the median survival time, were significantly longer in BCAA-treated patients than the control group [88]. This could be explained by the fact that plasma levels of BCAA decreased in patients with cirrhosis—a frequent underlying disease of HCC, leading to energy-protein malnutrition and impaired glycolysis and glycogenesis [89]. Many investigators reported the usefulness of BCAA supplementation in the treatment of cirrhosis and HCC [90,91,92]. However, the stimulation of protein synthesis by BCAAs—particularly leucine—could imply signaling pathways including mTOR [93]. Consequently, a large supplementation of BCAAs may potentially negatively impact tumor growth, as an undesirable side effect [94]. Hence, even if BCAA supplementation is probably useful for maintaining hepatic functional reserve in HCC patients [95], further studies are required to clarify the impact of BCAAs on protein balance and tumor growth to define the correct dosage of BCAAs supplementation.

β-hydroxy β-methyl butyrate (HMB) supplementation might be also a potential nutritional strategy to counteract the loss of muscle mass. A study [96] enrolled 472 patients with cancer which were supplemented by a mixture of HMB, glutamine (GLN), and arginine (ARG) or by an isonitrogenous, isocaloric control showing a strong trend towards higher fat-free mass in HMB/ARG/GLN-supplemented patients compared with controls. However, to date, studies assessing the impact of personalized nutritional strategies on muscle mass and clinical outcomes in cancer patients are very limited, and still lacking in patients treated with TKI therapy. 

## 6. Conclusions 

In sum, skeletal muscle changes have been observed during axitinib, lenvatinib, pazopanib, regorafenib, sorafenib, sunitinib, and vandetanib treatments. For all other TKIs, to our knowledge, no data are available, and studies are required to understand the impact of TKI therapy on muscle mass. Although the evidence remains to be confirmed, this review suggests that the loss of skeletal muscle mass may be exacerbated by different TKI treatments—such as axitinib, lenvatinib, regorafenib, sorafenib, or sunitinib—and could be associated with worse clinical outcomes in a wide range of cancers. Thus, the measurement of muscle mass from CT imaging could be helpful in predicting tolerance to TKI therapy and prognosis. In this context, every effort should be made to attenuate muscle wasting through early recognition of the loss of muscle mass and effective personalized nutritional support. Future clinical trials are needed to find the optimal nutritional support to countermeasure muscle mass depletion during TKI therapy.

## Figures and Tables

**Table 1:** Studies assessing the effect of TKI treatment on skeletal muscle mass.

TKI Treatment	First Author, Year of Publication, Country	Study Design	Cancer Type	Sample Size	Gender (M/F)	Age (Years) Median (IQR) or Mean ± SD	% Sarcopenic * Patients at Baseline	Method of Muscle Mass Assessment	Outcomes	Comparison	Mean ± SD or Median or %	*p*-Value
Axitinib	Chéry, 2017, USA [26]	R	Advanced non-metastatic RCC	24	18/5	60 (51.5–66)	30.4	L3 SMI (cm^2^/m^2^) calculated from CT scan	Median (IQR)	Baseline vs. 12 weeks after starting treatment	−2.9 (1.7–6.2)	<0.001
Lenvatinib	Rinninella, 2019, Italy [27]	CR	Advanced HCC	2	2/0	61 case 1 83 case 2	NR	L3 SMA (cm^2^) measured from CT scan	% SMA changes	Baseline vs. 24 months after starting treatment	Case 1: −2.13 Case 2: −10.83	NA
Lenvatinib	Hiraoka, 2019, Japan [28]	R	Advanced HCC	51	59/18	72.0 ± 8.9	NR	PI (cm^2^/m^2^) calculated from CT scan	Mean difference ± SD	Baseline vs. 4 weeks after starting treatment	−0.210 ± 0.315	NR
										Baseline vs. 12 weeks after starting treatment	−0.275 ± 0.372	NR
Regorafenib	Huemer, 2019, Austria [29]	R	Metastatic colorectal cancer	22	11/11	59 (42–74)	54	L3 SMI (cm^2^/m^2^) measured from CT scan	Mean difference	Baseline vs. after initiating treatment	−2.75 ± NR	<0.0001
Regorafenib	Bekir, 2020, Turkey [30]	R	Metastatic colorectal cancer	36	18/18	62 (52–69)	NR	L3 SMA (cm^2^) calculated from CT scan	Median (IQR)	Baseline vs. after initiating treatment	−7.8 (−13.9; −4.8)	0.001
Sorafenib	Antoun, 2010, France [31]	RCT	Advanced RCC	80	60/20	59.8 (38–78)	52.5	L3 SMA (cm^2^) calculated from CT scan	% SMA changes	Baseline vs. 6 months after starting treatment	−4.9	<0.01
									% SMA changes	Baseline vs. 12 months after starting treatment	−8	<0.01
									Mean difference ± SD	Placebo vs. sorafenib groups at month-6	−3.1 ± 1.3 vs. −7.4 ± 1.7	0.02
Sorafenib	Huillard, 2019, France [32]	R	Advanced differentiated thyroid cancer	365	NR	63 (24–82)	49.4	LBM (kg) estimated from L3 SMI calculated from CT scan	Mean difference	Placebo vs. sorafenib groups at month-6 after starting treatment	−0.1 vs. −3.0	<0.0001
Sorafenib or lenvatinib	Uchikawa, 2020, Japan [33]	R	Advanced HCC	67 (49/18)	56/11	70 (20–87)	NR	L3 SMI (cm^2^/m^2^) calculated from CT scan	Median	Before TKI treatment vs. 1–3 months afterward	45.3 (before TKI treatment) 42.1 (after treatment)	≤0.01^(1)^ 0.025^(2)^
Sunitinib or Sorafenib or others	Gu, 2017, China [34]	R	Metastatic RCC	101 (30/45/26)	65/36	59.6 ± 12.8	35.6	L3 SMI (cm^2^/m^2^)	Mean difference	Baseline vs. 4 months after starting treatment	−1.7 ± NR	NR
								Skeletal muscle density (HU) was calculated from CT scan.	Mean difference	Baseline vs. 4 months after starting treatment	−1.9 ± NR	R
Sunitinib or Pazopanib	Köstek, 2019, Turkey [35]	R	Metastatic RCC	36 (18/18)	25/11	60 (49–68)	NR	LBM (kg) estimated from L3 SMA calculated from CT scan	Median change (IQR)	Baseline vs. 4 months after starting sunitinib treatment	−5.6 (−1.2; −10.1)	0.02
										Baseline vs. 4 months after starting pazopanib treatment	−0.3 (−4.1; −1.0)	NS
Vandetanib	Massicotte, 2013, France [36]	Controlled trial	Advanced medullary thyroid carcinoma	23	16/7	51 (27–69)	NR	L3 SMI (cm^2^/m^2^) calculated from CT scan	Mean difference ± SD	Placebo vs. vandetanib group at month-3 after starting treatment	−1.0 ± 2.0 vs. 1.3 ± 2.1	0.009

Abbreviations: * sarcopenia was defined as SMI < sex-specific cut-off values. ^(1)^ sorafenib; ^(2)^ lenvatinib; CR, case report; CT, computed tomography; F, female; HCC, hepatocellular carcinoma; HU, Hounsfield unit; IQR, interquartile range; L3, third lumbar; LBM, lean body mass; M, male; NA, not applicable; NR, not reported; NS, not significant; RCC, renal cell carcinoma; RCT, randomized controlled trial; R, retrospective; SD, standard deviation; SMA, skeletal muscle area; SMI, skeletal muscle index; TKI, tyrosine kinase inhibitor; vs. versus.
